# Inhibiting USP8 overcomes hepatocellular carcinoma resistance via suppressing receptor tyrosine kinases

**DOI:** 10.18632/aging.203061

**Published:** 2021-06-03

**Authors:** Ying Zhu, Jianguo Xu, Wei Hu, Fang Wang, Yan Zhou, Wei Gong, Wen Xu

**Affiliations:** 1 Department of Gastroenterology, Shenzhen Hospital of Southern Medical University, Shenzhen, Guangdong, China; 2 Department of Liver Disease Center, Shenzhen Hospital of Southern Medical University, Shenzhen, Guangdong, China; 3 Information Management Section, Bethune International Peace Hospital, Shijiazhuang, Hebei Province, China

**Keywords:** USP8, receptor tyrosine kinase, HCC, chemoresistance

## Abstract

The ubiquitin-specific protease 8 (USP8) is a prototypic multidomain deubiquitinating enzyme with pleiotropic functions. We investigated the role of USP8 in hepatocellular carcinoma (HCC) by analyzing expression patterns of USP8 in HCC patients, and evaluating its functions and underlying signaling. Among 20 HCC patients investigated, we found that USP8 protein upregulation was a common phenomenon (17 out of 20) in HCC compared to normal liver tissue. Furthermore, the upregulation of USP8 was not associated with any clinicopathology. USP8 inhibition via genetic and pharmacological approaches resulted in growth inhibition and apoptosis induction in both sensitive and doxorubicin-resistant HCC cells. Of note, USP8 inhibition significantly enhanced doxorubicin or sorafenib’s efficacy in HCC cells and mouse models. We further found that USP8 inhibition decreased levels of multiple receptor tyrosine kinases (RTKs) by ~90%, such as epidermal growth factor receptor (EGFR) and c-Met. Consistently, the downstream signaling regulated by RTKs was disrupted in HCC cells after USP8 inhibition, as shown by the decreased p-Akt, p-STAT3 and p-Raf. Our findings demonstrate that USP8 is a novel therapeutic target in HCC. Inhibiting USP8 has potential to overcome current drug resistance, particularly on HCC patients with high USP8 expression.

## INTRODUCTION

Hepatocellular carcinoma (HCC) is third most common cause of cancer death in China, and its incidence is gradually increasing [1]. Majority of HCC patients are attributed to hepatitis B virus (HBV) or hepatitis C virus (HCV) infections. Only patients with early stage cancer are eligible for potentially curative therapies, such as surgical removal and liver transplantation [2]. For two-thirds of newly diagnosed HCC patients at intermediate or advanced stages, chemoembolization and sorafenib are standard of care treatments [3, 4]. HCC depends on activation of pathways involving tyrosine kinase receptors (TKR), including epidermal growth factor receptor (EGFR), vascular endothelial growth factor receptor (VEGFR), and hepatocyte growth factor (HGF)/c-mesenchymal-epithelial transition factor (c-Met), to activate Ras/Raf/mitogen-activated protein Kinases (MAPK) and phosphatidylinositol 3-kinase (PI3K)/Akt/mammalian target of rapamycin (mTOR) signalling pathways that are important for proliferation, survival and angiogenesis [5]. Sorafenib is a multi-kinase inhibitor against Raf, VEGFR, c-Kit and platelet-derived growth factor receptor (PDGFR) [6].

Ubiquitination, a post-translational modification that promotes protein degradation, is regulated by both ubiquitin ligases and deubiquitinases (DUBs). Ubiquitin-specific proteases (USPs) is the largest DUB subfamily, and their dysregulation correlates with tumorigenesis, cancer progression and other diseases [7–10]. USP8 is a member of the USPs family, and its role in tumors has been recently identified. High expression of USP8 is associated with poor prognosis in patients with cervical cancer and early-stage lung adenocarcinoma [11, 12]. Inhibition of USP8 suppresses growth and invasion of cholangiocarcinoma and corticotroph adenomas [13, 14]. Studies further reveal that the anti-cancer potential of USP8 inhibition is due to downregulation of several TKRs [14, 15]. In this study, we systematically evaluated expression patterns of USP8 in HCC patients and associated functions in sensitive and resistant HCC cell lines. We also addressed the molecular underlying mechanisms of USP8 inhibition in HCC cells.

## MATERIALS AND METHODS

### Clinical samples and ethics statement

This study was approved by the ethics committee of Shenzhen Hospital of Southern Medical University. HCC and corresponding adjacent normal livers were obtained from patients during surgeries, and were handled following ethical and legal standards. Written informed consent was obtained from all enrolled patients.

### Cell culture and generation of doxorubicin-resistant lines

Parental HuH6 and HepG2 cell lines (ATCC) were cultured in RPMI-1640 medium supplemented with 10% fetal bovine serum (FBS; Gibco), penicillin/streptomycin (Life Technologies). Doxorubicin-resistant cell lines HuH6-r and HepG2-r were established as previously described [16]. Briefly, parental cells were cultured in presence of doxorubicin for ~ 6 months. The concentration of doxorubicin was gradually increased by 1.5- to 2-fold after 2-3 weeks of culturing for stable cell proliferation. Established resistant cells were maintained in the presence of 5 μM doxorubicin.

### Drugs and antibodies

Doxorubicin (Sigma-Aldrich), USP8 inhibitor (9-ehtyloxyimino9H-indeno [1,2-b] pyrazine-2,3-dicarbonitrile; Melone Pharmaceutical) and sorafenib (Selleckchem) were reconstituted in dimethyl sulfoxide (DMSO; Sigma-Aldrich). Antibodies to detect phosphorylated c-Met, EGFR, Akt, Stat3 and Raf and their corresponding total protein levels were obtained from Cell Signaling. Antibodies to detect USP8 and β-actin were obtained from Sigma-Aldrich and Santa Cruz, respectively.

### Immunohistochemical analysis

The frozen tissue specimens were sectioned, placed onto glass slides and fixed with 4% paraformaldehyde. The sections were incubated in 200x diluted USP8 antibody solution overnight at 4° C. The next day, sections were incubated in anti-Rabbit IgG, HRP-linked antibody solution, followed by DAB (3, 3-diaminobenzidine) staining. Hematoxylin was applied as a counterstain.

### Proliferation and apoptosis assays

Cells were seeded up to 70% confluence on plates for proliferation and apoptosis assays using the same methods as described previously [16]. Briefly, cell proliferation was evaluated using BrdU Proliferation Assay Kit (Cell Signaling). Cell apoptosis was determined using flow cytometry with Annexin V-FITC and 7-AAD (BD Pharmingen) staining. Detailed experimental conditions were indicated in figure legends.

### USP8 ELISA assay using cell and tissue samples

Cellular USP8 level was quantified using USP8 ELISA kit (Aviva Systems Biology) according to manufacturer’s instructions. Briefly, cells or tissues were harvested and homogenized using a standard protocol, and samples were adjusted to the same concentration using reagents provided in the kit. Samples were added to the USP8 antibody pre-coated wells, followed by incubation, washing, addition of conjugate and substrate. Absorbance was read at 450 nm on the microplate reader (Fisher Scientific).

### Transfection

siRNA targeting USP8 were transfected into cells using Dharmafect Transfection Reagent as per manufacturer’s protocols. USP8 siRNA sequences were the same as previously described [12]. USP8 siRNA and scramble siRNA (si-Ctrl) were purchased from GenePharma. USP8 levels were examined after 48 h post-transfection.

### Immunoblotting

Proteins were extracted using RIPA buffer and concentrations were determined using BCA protein assay kit (Pierce). Equal amounts of proteins were loaded to the SDS-PAGE gel, followed by electrophoresis. Immunoblotting was performed using a standard protocol as described previously [17].

### HCC xenograft model

All animal handling procedures were conducted in accordance with animal care guidelines provided by the Southern Medical University (Shenzhen, China). Male SCID mice at 4-6-weeks-old were obtained from Hunan SJA Laboratory Animal Co., Ltd and housed in a pathogen-free environment with 12-hour light/dark cycles. To generate doxorubicin -sensitive and -resistant HCC cell models, ten million HepG2 and HepG2-r cells in 100 μl of PBS were implanted subcutaneously in the hind flank of each mouse. After developing palpable tumors, the mice were randomly grouped for drug treatment. The specific drug dose and administration routes were indicated in figure legends. Tumor length and width were measured every 5 days, and associated tumor volumes were determined using a standard formula.

### Statistical analyses

Statistical analyses of differences between two groups were performed using one-way analysis of variance (ANOVA) and subsequently by unpaired Student’s t test. *P* value < 0.01 is considered statistically significant.

## RESULTS

### Upregulation of USP8 expression is common among HCC patients

**Figure 1:**
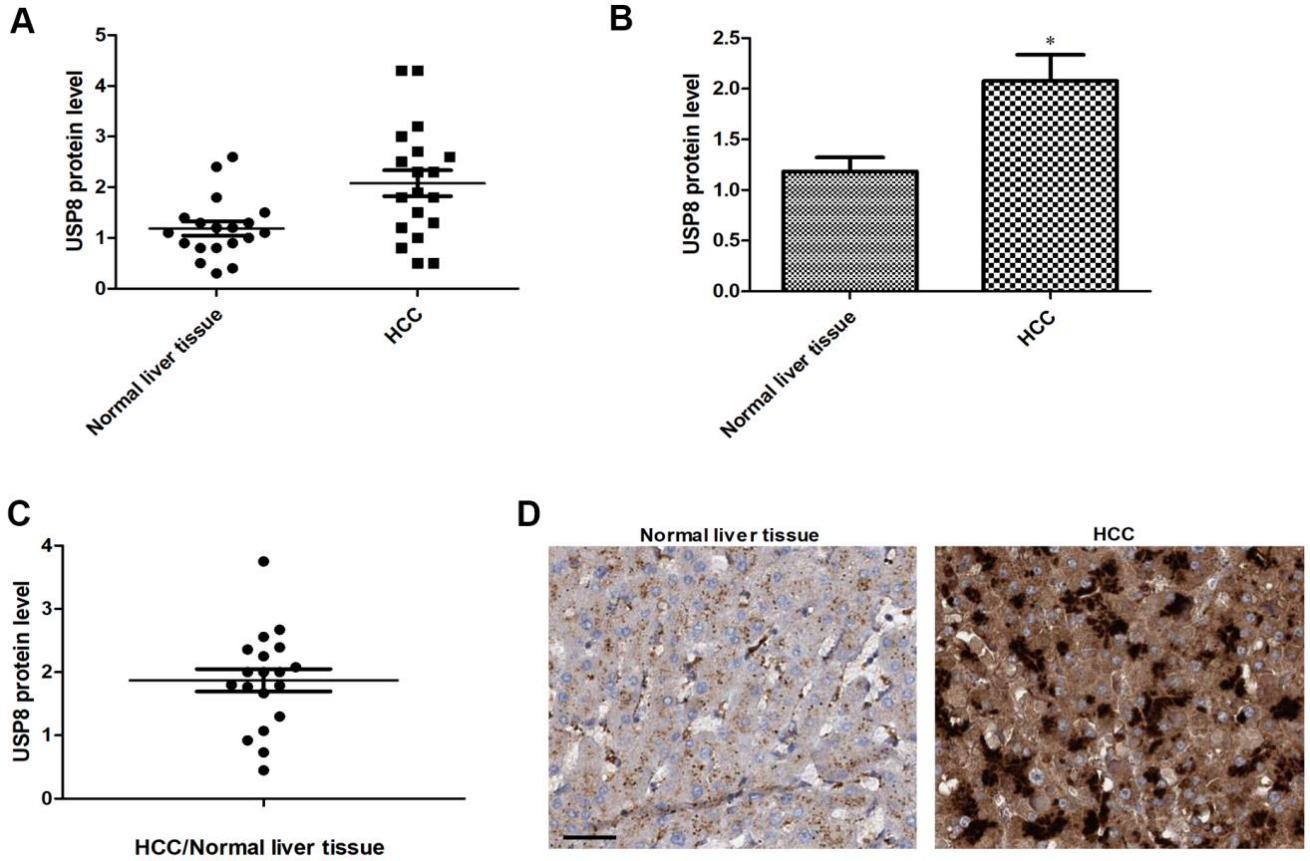
**USP8 expression is upregulated in HCC patients.** (**A**) Scatter plot of USP8 protein level in individual HCC and normal liver (n=20). (**B**) The average level of USP8 protein in HCC and normal liver (n=20). (**C**) USP8 protein ratio of tumor and adjacent normal in individual HCC patients (n=20). (**D**) Representative images of immunohistochemical staining for USP8 in paired normal liver and HCC. Nuclear is stained with hematoxylin. Scale bar is 10 μM. USP8 protein level was normalized with total protein amount. *, p<0.01, compared to normal liver.

We first analyzed expression patterns of USP8 in HCC and compared that with results from matched normal liver tissue obtained from same patients. In order to quantify USP8 level in a complex biological sample and make fair comparison, we conducted an enzyme-linked immunosorbent assay (ELISA) that used specific USP8 antibody to bind and measure. We analyzed twenty HCC patients with clinicopathological information shown in Supplementary Table 1. We found that USP8 expressions varied among both normal liver and HCC, and there were up to 4-folds and 3-folds differences between the highest and lowest in HCC and normal liver respectively (Figure 1A). The average level of USP8 was significantly higher in HCC compared to normal counterparts, with an observed 2-folds increase (Figure 1B). Specifically, 17 out of 20 patients displayed ratios of USP8 protein level of HCC/normal liver >1, indicating that USP8 expression is upregulated in majority of HCC patients. Consistent with ELISA results, a representative immunohistochemistry analysis of HCC and normal liver from one patient demonstrated a higher USP8 staining in HCC than normal liver tissues (Figure 1D). In addition, we did not observe any association of USP8 expression with patients’ clinicopathology, such as disease stage and virus infection status (Supplementary Table 1).

### USP8 knockdown is active against sensitive and resistant HCC cells

**Figure 2:**
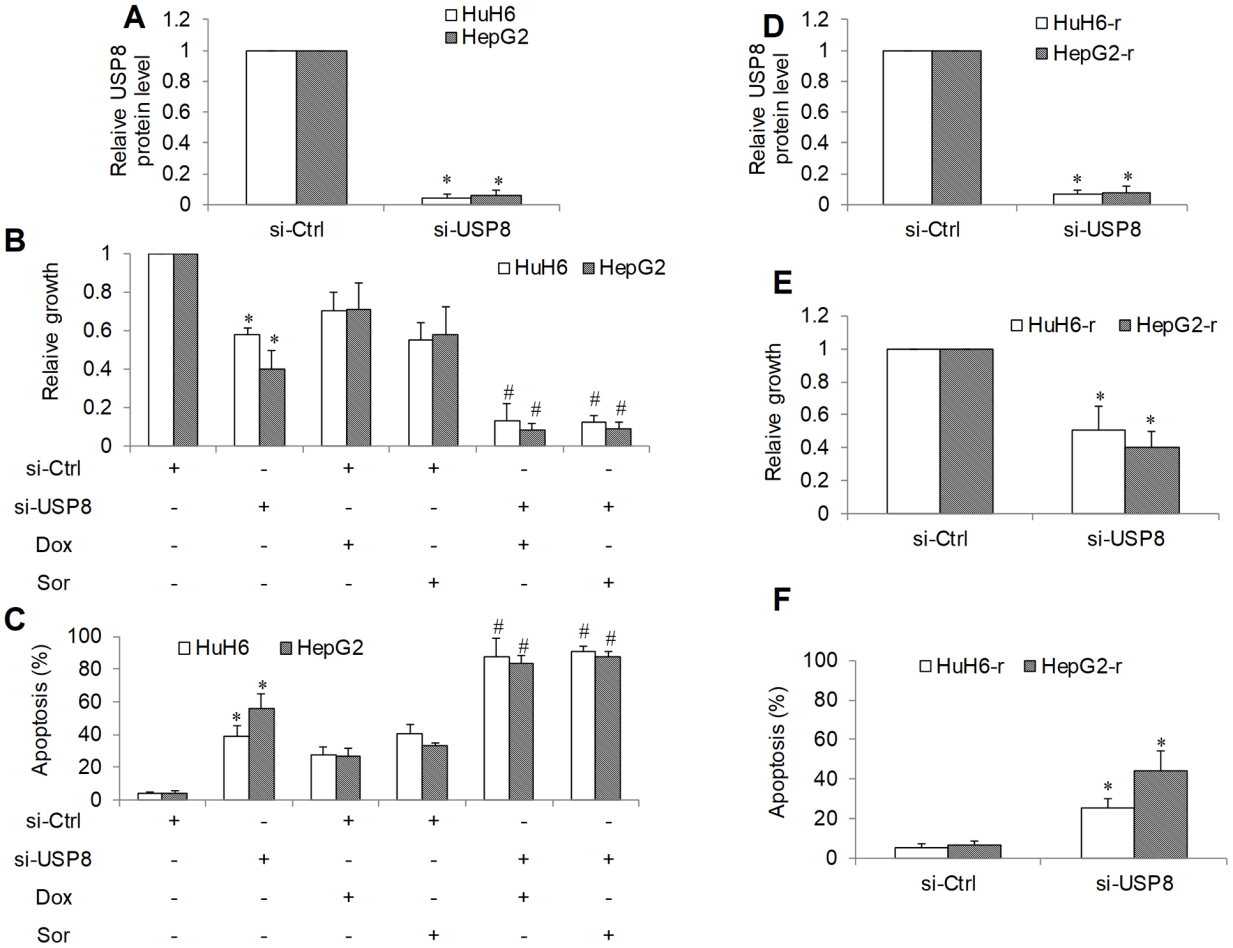
**The inhibitory effects of genetic knockdown of USP8 in HCC.** (**A**) USP8 protein level in USP8-depleted HCC cells. USP8 knockdown inhibits proliferation (**B**) and induces apoptosis (**C**) in HuH6 and HepG2 cells, and significantly enhances the efficacy of doxorubicin (Dox) and sorafenib (Sor). (**D**) USP8 protein level in USP8-depleted doxorubicin-resistant HCC cells. USP8 knockdown inhibits proliferation (**E**) and induces apoptosis (**F**) in HuH6-r and HepG2-r cells. Doxorubicin at 0.1 μM and sorafenib at 0.5 μM were used. Cells were harvested for USP8 protein level analysis at 48 hours post-transfection. Drugs were added to cells at 48 hours post-transfection. Cell proliferation and apoptosis were determined after 3 days treatment. *p<0.01, compared to control. #p<0.01, compared to doxorubicin or sorafenib alone.

We next depleted USP8 gene expression using siRNA knockdown approach in two HCC cell lines and confirmed that there was minimal level of USP8 in si-USP8 cells (Figure 2A and Supplementary Figure 1A). We further observed an ~40% and ~60% cell proliferation reduction as assessed by measuring BrdU level (Figure 2B). Correspondingly, ~40% and ~55% cell apoptosis was shown with flow cytometry of Annexin V (Figure 2C) in HuH6 and HepG2 cells, respectively. We added standard of care drugs (eg, doxorubicin and sorafenib [18, 19]) to USP8-depleted cells and measured proliferation and apoptosis. Doxorubicin and sorafenib alone at the tested concentration inhibited ~30% to ~50% proliferation and induced ~20% to ~40% apoptosis in si-Ctrl HCC cells (Figure 2B, 2C and Supplementary Figures 2, 3). We found that doxorubicin and sorafenib were significantly more effective in USP8-depleted cells, resulting in ~80% proliferation reduction and 90% apoptosis induction. These results suggest that USP8 inhibition is active against HCC cells resistant to doxorubicin or sorafenib. To support this, we depleted USP8 in two well-established doxorubicin-resistant HCC lines: HuH6-r and HepG2-r [16, 19] and confirmed the efficacy of siRNA knockdown (Figure 2D and Supplementary Figure 1B). USP8 knockdown also effectively inhibited proliferation (~50% to ~70%) and induced apoptosis (~20% to ~40%) in doxorubicin-resistant HCC cell lines (Figure 2E, 2F and Supplementary Figures 4, 5). We noted that extent of USP8 inhibition on growth and survival were similar between sensitive and resistant cells, demonstrating that USP8 knockdown is active against both sensitive and resistant HCC.

### USP8 inhibitor is active against sensitive and resistant HCC cells *in vitro* and *in vivo*

**Figure 3:**
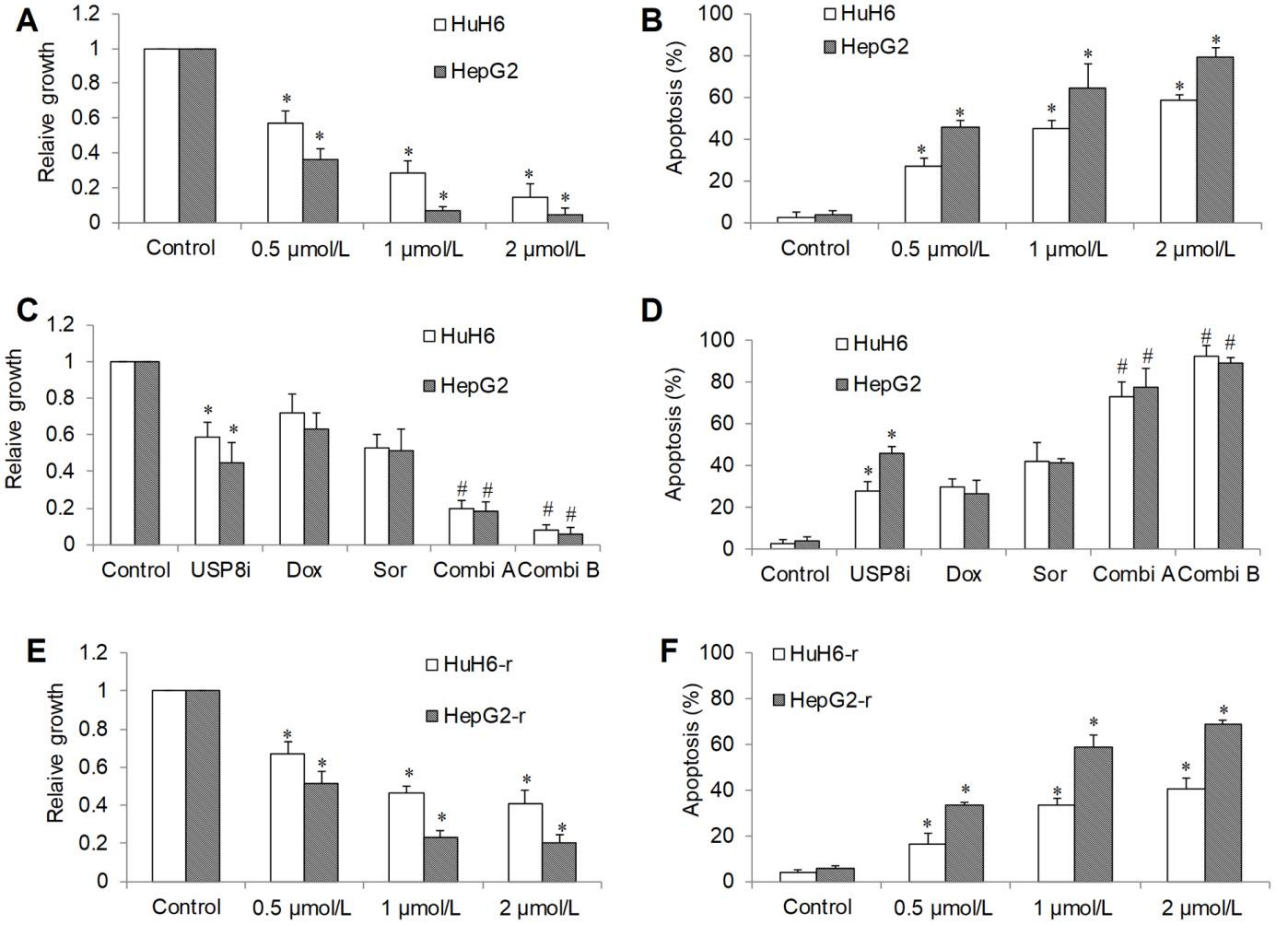
**The inhibitory effects of pharmacological inhibition of USP8 in HCC *in vitro*.** (**A**) USP8 inhibitor (USP8i) at 0.5, 1 and 2 μmol/L significantly inhibits growth (**A**) and induces apoptosis (**B**) in HCC cells. Combination of USP8i with doxorubicin or sorafenib further significantly inhibits further proliferation (**C**) and induces more apoptosis (**D**) in HCC cells than single drug alone. USP8i inhibits proliferation (**E**) and induces apoptosis (**F**) in HuH6-r and HepG2-r cells. Doxorubicin at 0.1 μM and sorafenib at 0.5 μM were used. Doxorubicin and sorafenib were used in Combi1 and Combi2, respectively. Cell proliferation and apoptosis were determined after 3 days treatment. *p<0.01, compared to control. #p<0.01, compared to doxorubicin or sorafenib alone.

In order to investigate the translational potential of targeting USP8 in HCC, we tested the efficacy of a specific USP8 inhibitor: USP8i (9-ehtyloxyimino9H-indeno[1,2-b] pyrazine-2,3-dicarbonitrile) [13, 20]. Consistent with USP8 knockdown, we found that USP8i at low micromolar range dose-dependently inhibited proliferation and induced apoptosis (Figure 3A, 3B). Combination of USP8i with sorafenib or doxorubicin inhibited significantly more proliferation and induced significantly higher rate of apoptosis in HCC cells (Figure 3C, 3D). Similar to USP8 knockdown, the combination of sorafenib or doxorubicin with USP8i led to nearly complete inhibition of growth and survival. USP8i also dose-dependently inhibited proliferation and induced apoptosis in doxorubicin-resistant HCC cells. In addition, HepG2-r cells seemed to be more sensitive to USP8i than HuH6-r cells.

**Figure 4:**
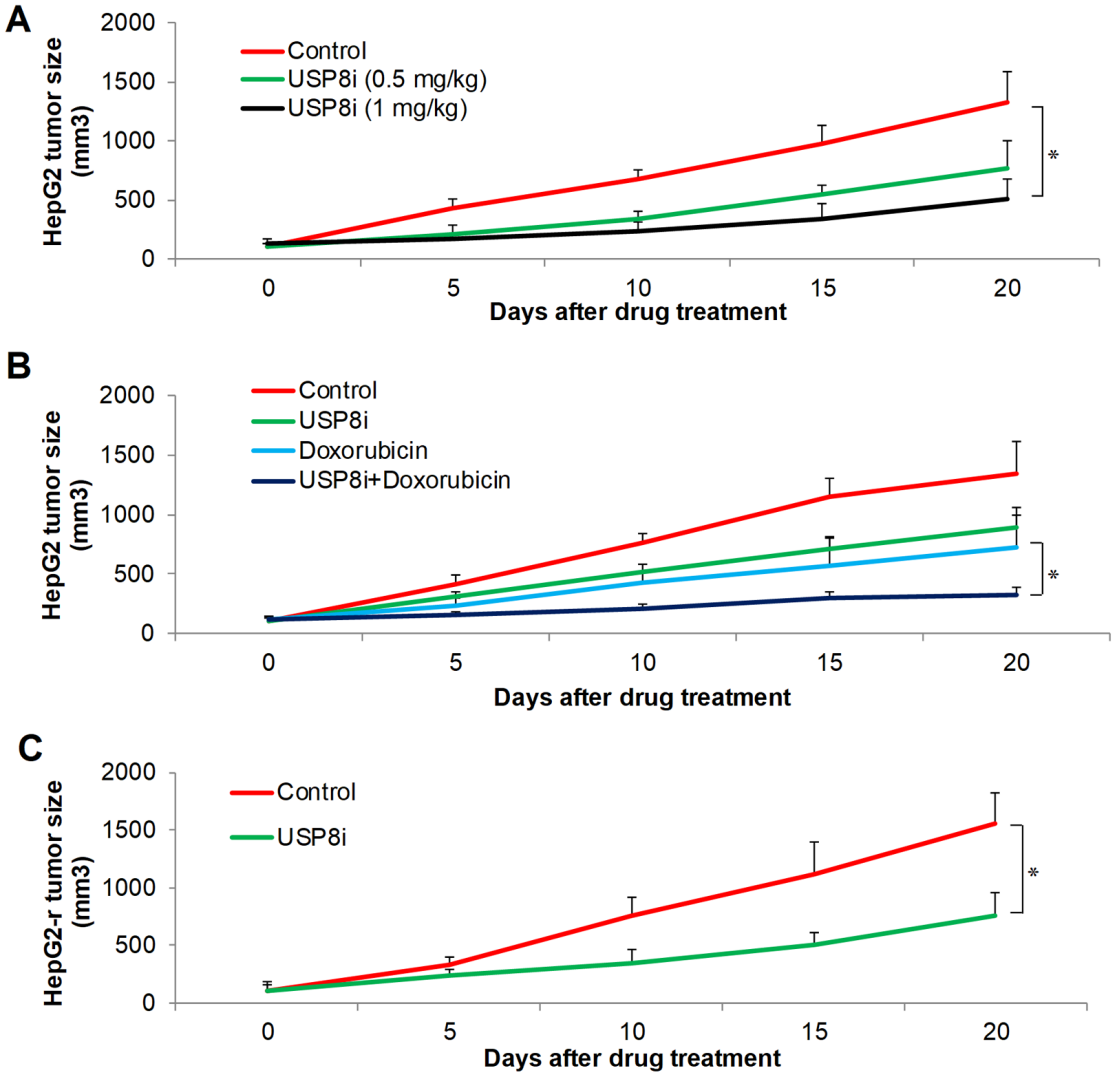
**The inhibitory effects of pharmacological inhibition of USP8 in HCC *in vivo*.** (**A**) USP8i significantly inhibits HepG2 growth in mice. Mice were treated with vehicle alone, USP8i (0.5 mg/kg or 1 mg/kg once per day, intraperitoneal injection). (**B**) Combination of USP8i and doxorubicin is superior than in inhibiting HepG2 growth in mice. Mice were treated with vehicle alone, USP8i (0.5 mg/kg once per day, intraperitoneal injection), doxorubicin (4 mg/kg once per five days, intraperitoneal injection,), or a combination of doxorubicin and USP8i. (**C**) USP8i significantly inhibited doxorubicin-resistant HepG2-r growth in mice. Mice were treated with vehicle alone, USP8i (0.5 mg/kg once per day, intraperitoneal injection). *p<0.01, compared to control or doxorubicin.

We further tested *in vivo* efficacy of USP8i alone and combinatory efficacy of USP8i with doxorubicin on HCC xenograft mouse model. Consistent with *in vitro* observations, USP8i at 0.5 mg/kg and 1 mg/kg significantly inhibited HCC growth in mice in a dose-dependent manner (Figure 4A). In addition, the mice tolerated all treatments well, except a slight body weight loss registered in mice receiving 1 mg/kg USP8i (Supplementary Figure 6). Of note, the combination of USP8i and doxorubicin was more effective than single drug alone in inhibiting HCC growth *in vivo* (Figure 4B). Consistently, USP8i at 0.5 mg/kg also significantly inhibited growth of doxorubicin-resistant HCC in mice (Figure 4C).

### USP8 inhibition downregulates oncogenic receptor tyrosine kinase expression and signaling in HCC cells

**Figure 5:**
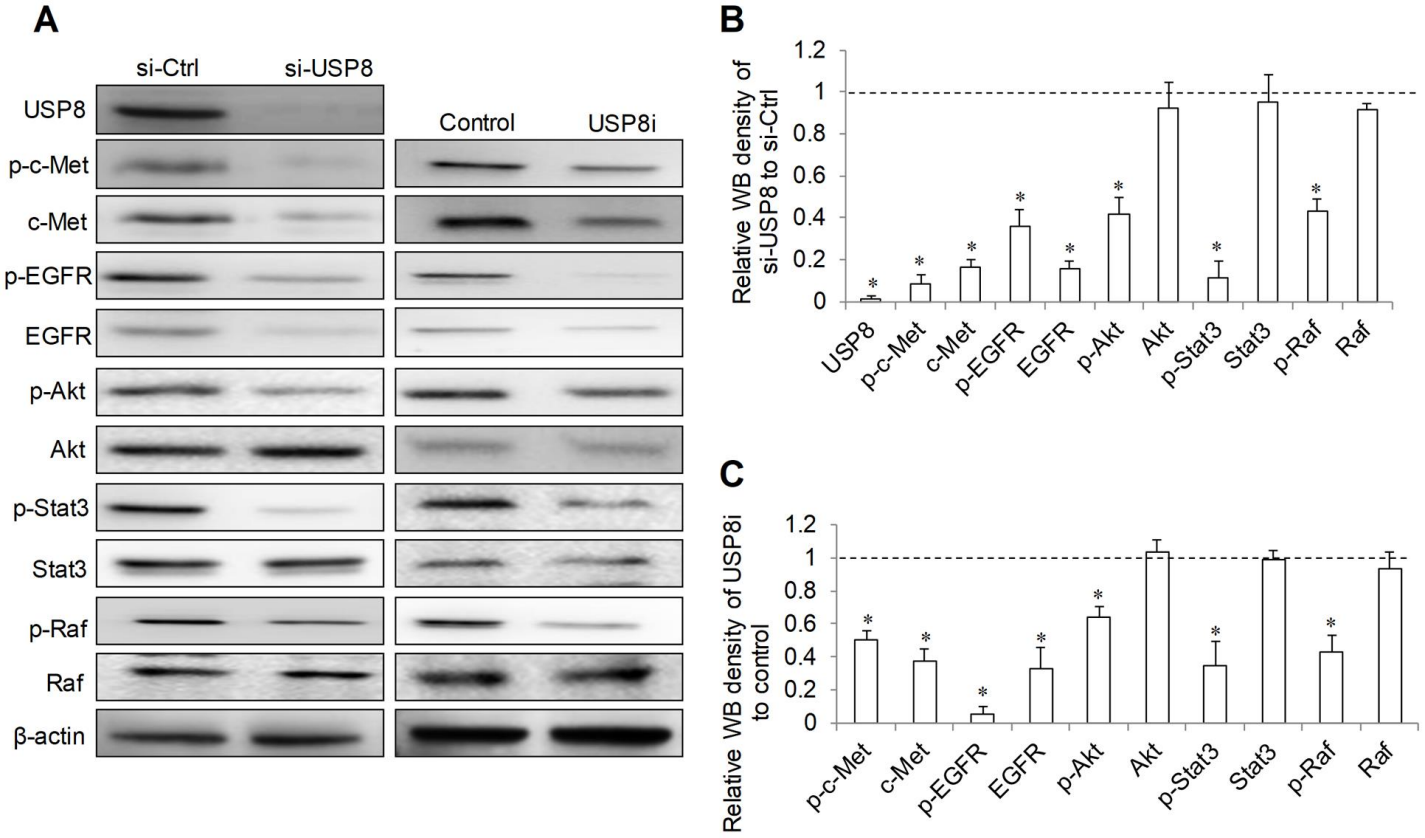
**Effect of USP8 inhibition on receptor tyrosine kinase signaling in HCC cells.** Representative image (**A**) and quantification (**B**, **C**) of western blots analysis of p-c-Met(Y1234/1235), c-Met, p-EGFR(Y1086), EGFR, p-Akt (S437), Akt, p-Stat3(T705), Stat3, p-Raf (S338/T341) and Raf in HuH6 cells after USP8 inhibition. si-Ctrl and control value were set as 1. Western blot was analysed after 24-hour drug treatment in HuH6 cells or at 72-hour post-transfection in siRNA transfected cells. *p<0.01, compared to si-Ctrl or control.

USP8 inhibition has been shown to downregulate several receptor tyrosine kinases including ErbB2, c-Met and EGFR [13]. Given the importance of EGF/EGFR and HGF/Met signaling in HCC progression and drug resistance [5], we performed immunoblotting analysis to evaluate the level of phosphor- and total EGFR and -c-Met in HCC cells after USP8 inhibition. We observed the remarkable reduction in p-EGFR at Y1086 and p-c-Met at Y1234/1235 in USP8-depleted cells (si-USP8; Figure 5A). Total EGFR and c-Met were also reduced in si-USP8 cells. Densitometry analysis of Western immunoblotting demonstrated that there were ~90%, ~80%, ~60% and ~80% reduction of p-c-Met, c-Met, p-EGFR and EGFR in si-USP8 cells respectively (Figure 5B). Consistently, USP8 inhibition by USP8i decreased p-EGFR (~90%), EGFR (~70%), p-c-Met (~50%) and c-Met (~60%) in HCC cells respectively (Figure 5A, 5C). Raf/MER/ERK, PI3K/Akt/mTOR and Jak/STAT are known downstream pathways regulated by receptor tyrosine kinases [5]. We further analysed the molecules involved in these signalling pathways. We found that USP8 inhibition via siRNA or pharmacological inhibitor significantly decreased p-Akt (~40% to ~60%), p-Raf (~60%) and p-STAT3 (~60% to ~80%) without affecting their corresponding total proteins (Figure 5). Our results clearly demonstrate that USP8 inhibition downregulates oncogenic receptor tyrosine kinase expression and signalling in HCC cells.

## DISCUSSION

The clinical management of advanced HCC is still challenging due to high refractoriness to standard of care, including chemotherapy and molecular targeted therapy [21]. In the past decades, many research efforts have been focused on understanding underlying mechanisms responsible for drug resistance and developing novel therapeutic agents which could potentially synergize existing treatments. We previously identified that prenylation, a post-translational modification process required by many oncogenic proteins, contribute to HCC progression and drug resistance [16]. In this work, we investigated the role of USP8 in HCC growth and survival. USP8 is a member of USPs family that regulate ubiquitination, another post-translational modification serving to ensure cell homeostasis [22]. Aberrant ubiquitination alters biological processes that can induce cancer, and targeting ubiquitination via inhibiting proteasome has achieved tangible success for treatment of cancer, such as multiple myeloma [23]. Increasing evidence demonstrates that USPs promote progression of many cancers, including HCC, via stabilizing c-Myc, regulating apoptosis-related factors, cancer stemness and microenvironment [8]. For example, aberrant activations of USP11, USP5 and USP22 have been reported to promote tumorigenesis and activates proliferation, metastasis and multidrug resistance in HCC [24–27]. The role of USP8 in HCC is unknown.

Compared with normal counterparts, we found that USP8 upregulation was common in tumor tissues of HCC patients (17/20) (Figure 1). Aberrant upregulation of USP8 was also observed in other cancers, such as cervical squamous cell carcinoma and lung adenocarcinoma [11, 12]. In addition, USP8 expression was correlated with EGFR mutation status and patients who had USP8-positive lung adenocarcinoma had significantly poorer outcomes than those who were USP8-negative [12]. Consistently, high expression of USP8 was correlated with advanced tumor stage and high recurrence risk in cervical cancer patients [11]. Although our study demonstrated that USP8 upregulation was less likely to be correlated with gender, age, HBV or HCV infection and diseases stage (Supplementary Table 1), whether USP8 has prognostic value in HCC patients and its expression is correlated with oncogenes’ mutation status is worthy of further investigations in a large cohort. In contrast, USP8 was downregulated in breast cancer and patients in USP8 high-expression group were correlated with better clinical characteristics [28]. The contradictory observations on the USP8 expression pattern between normal and malignant tissues suggest that USP8 downregulation or upregulation is likely to be tumor type specific.

Our functional studies using both genetic and pharmacological approaches on two HCC cell lines confirmed that USP8 inhibition was effective to inhibit HCC proliferation and induce apoptosis (Figures 2A–2C, 3A, 3B). This is supported by previous reports emphasizing the important role of USP8 in cancer growth, survival and invasion [29]. We further demonstrated that USP8 inhibition can overcome HCC drug resistance as shown by the observations that USP8 inhibition 1) was active against doxorubicin-resistant HCC cells and 2) significantly augmented the inhibitory effects of doxorubicin and sorafenib (Figures 2D–2F, 3C–3F). Two studies reported that USP8 is a novel target for overcoming gefitinib resistance in lung cancer [13, 15]. Our work together with other reports confirm the potential of USP8 inhibition to overcome cancer resistance. This is important because drug resistance is the major hallmark in treatment failure for advanced HCC. Our work further demonstrates the therapeutic potential of targeting USP8 in HCC using USP8i. The results obtained from HCC xenograft mouse models clearly indicate the efficacy of USP8i as a single drug alone and its combination with doxorubicin in inhibiting HCC growth in mice (Figure 4). USP8i at dose that significantly enhanced efficacy of doxorubicin without causing toxicity in mice suggests the therapeutic window of USP8i for the treatment of HCC.

A significant finding in this work is that USP8 inhibition targets RTKs in HCC cells (Figure 5). We observed a remarkable reduction of total EGFR and c-Met as well as p-EGFR and p-c-Met by USP8 inhibition. HGF/c-Met is well known to promote HCC growth and metastasis [30]. EGFR overexpression is frequently observed in HCC and EGFR activation is a determinant of primary resistance of HCC cells to sorafenib [31]. Our work provides pre-clinical evidence that USP8 is an attractive therapeutic candidate as USP8 inhibition target both EGFR and c-Met in HCC. Raf/MEK/ERK, PI3K/Akt/mTOR and Jak/STAT pathways are downstream cell signaling of RTKs in HCC to drive cell survival, proliferation, migration, angiogenesis and metastasis [5]. Consistent with RTKs inhibition, we observed a significant reduction of p-Akt, p-STAT3 and p-Raf (Figure 5), confirming that USP8 inhibition targets multiple RTKs in HCC, which is in agreement with previous reports [13–15].

In conclusion, we demonstrate that USP8 upregulation is a molecular feature in ~80% HCC patients. USP8 inhibition is active against sensitive and resistant HCC cells, via downregulating multiple RTKs. These results suggest that USP8 is a novel therapeutic target to overcome HCC drug resistance, particularly in patients with high expression of USP8.

## Supplementary Material

Supplementary Figures

Supplementary Table 1
