# Supplementary material for: Inhibiting USP8 overcomes hepatocellular carcinoma resistance via suppressing receptor tyrosine kinases
Source: Aging (Albany NY). 2021 Jun 3;13(11):14999–5012. doi: 10.18632/aging.203061 (PMC8221339; doi:10.18632/aging.203061)
Supplement: Supplementary Figures [file aging-13-203061-s001.pdf]

SUPPLEMENTARY MATERIALS

Supplementary Figures

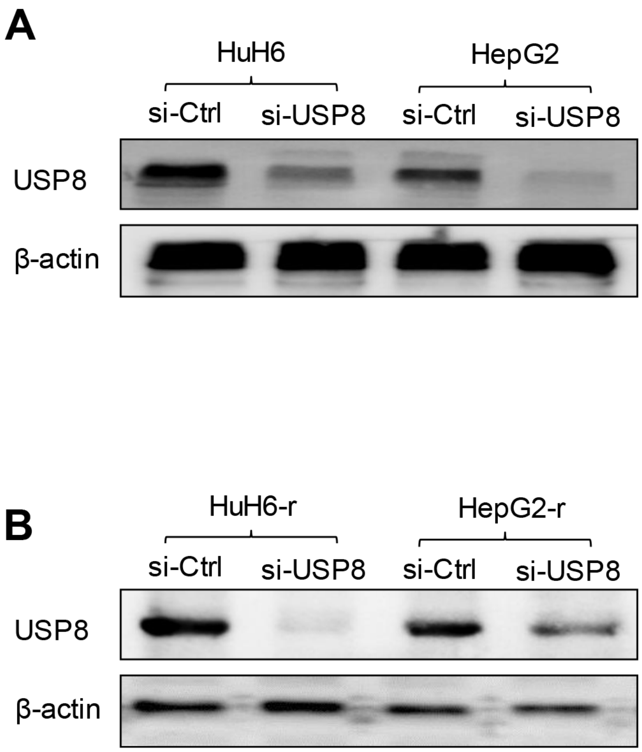

Supplementary Figure 1. Reduction of USP8 protein in si-USP8 treated HCC cell lines. Western blot of USP8 in HuH6 and HepG2 (**A**), HuH6-r and HepG2-r (**B**) cells after si-USP8 transfection.

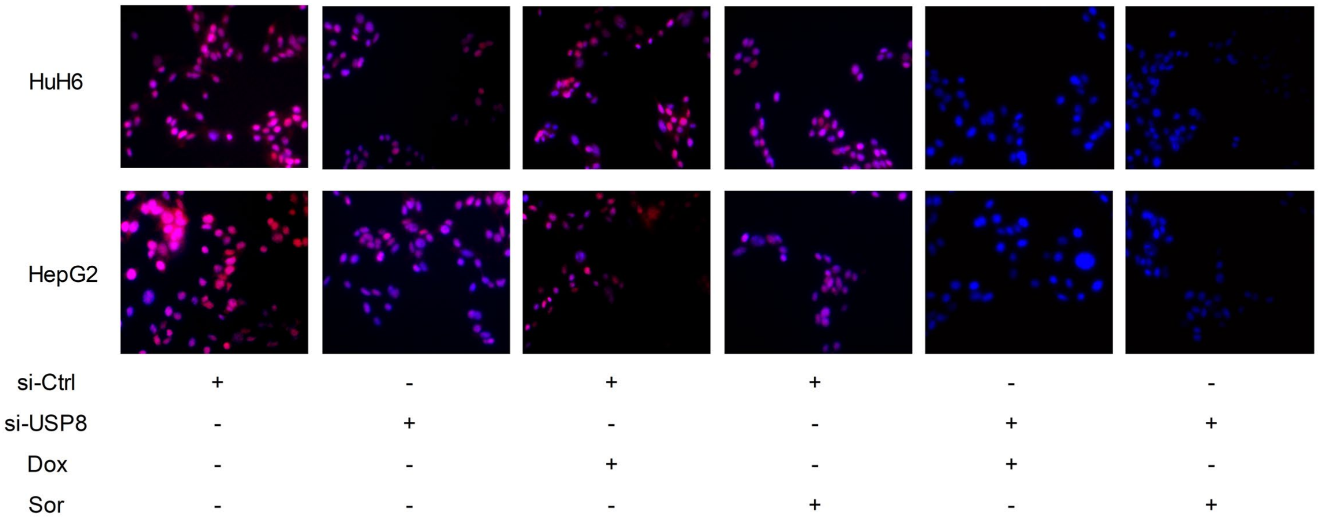

Supplementary Figure 2. Representative BrdU staining in si-USP8 treated HCC cell lines. Doxorubicin at 0.1 µM and sorafenib at 0.5 µM were added to cells at 48 hours post-transfection. BrdU staining was performed after 3 days drug treatment.

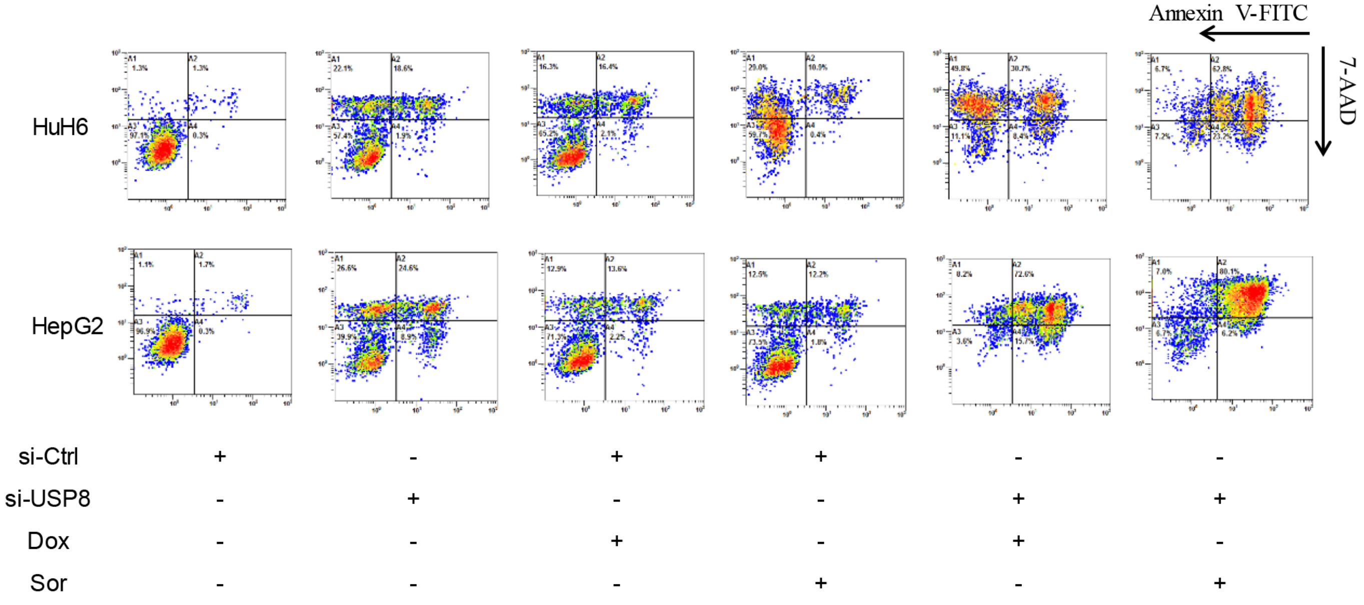

Supplementary Figure 3. Representative FACS images in si-USP8 treated HCC cell lines. Doxorubicin at 0.1 µM and sorafenib at 0.5 µM were added to cells at 48 hours post-transfection. Flow cytometry of Annexin V and 7-AAD staining was performed after 3 days drug treatment.

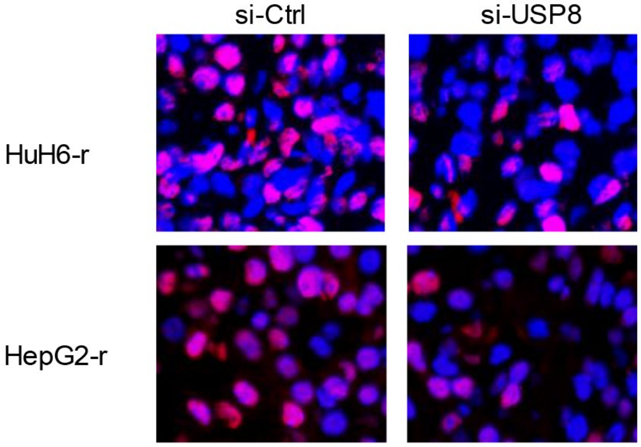

Supplementary Figure 4. Representative BrdU staining in si-USP8 treated HCC cell lines.

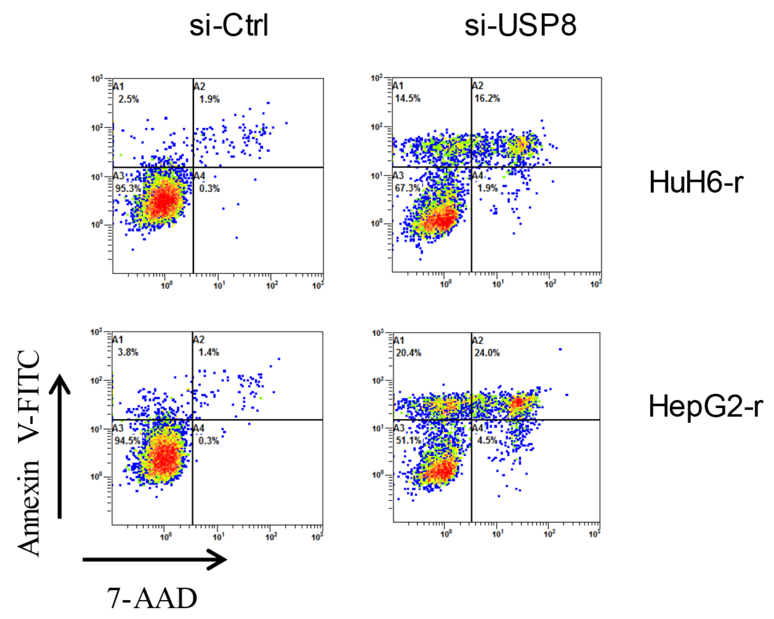

Supplementary Figure 5. Representative FACS images in si-USP8 treated HCC cell lines.

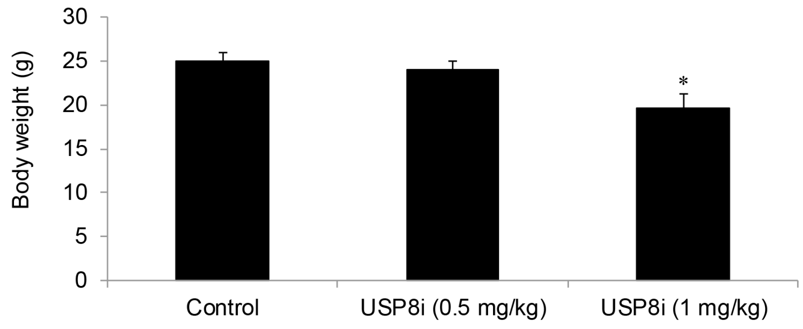

Supplementary Figure 6. Mice body weight in control and USP8i-treated groups. USP8i at 1 mg/kg but not 0.5 mg/kg significantly decreased mice body weight. *p<0.05, compared to control.
