# Supplementary material for: Inhibiting USP8 overcomes hepatocellular carcinoma resistance via suppressing receptor tyrosine kinases
Source: Aging (Albany NY). 2021 Jun 3;13(11):14999–5012. doi: 10.18632/aging.203061 (PMC8221339; doi:10.18632/aging.203061)
Supplement: Supplementary Table 1 [file aging-13-203061-s002.pdf]

SUPPLEMENTARY TABLE

Supplementary Table 1. HCC patients' information.

Patient number	Age	TNM stage	HBV	HCV	T/N ratio (USP8)
HCC#1	34	III	+	-	1.30
HCC#2	46	III	-	-	2.67
HCC#3	45	I	+		1.77
HCC#4	57	II	-	+	0.73
HCC#5	68	II	-	-	0.45
HCC#6	72	II	-	-	3.75
HCC#7	58	III	-	-	2.08
HCC#8	76	II	+	-	1.80
HCC#9	44	II	+	-	2.36
HCC#10	65	I	+	-	2.25
HCC#11	64	I	+	-	2.00
HCC#12	75	II	-	-	2.56
HCC#13	61	II	-	-	1.67
HCC#14	54	III	-	-	2.00
HCC#15	67	II	-	+	0.92
HCC#16	63	II	-	-	2.00
HCC#17	73	II	+	-	1.07
HCC#18	49	III	+	-	1.79
HCC#19	49	II	+	-	2.39
HCC#20	82	I	-	-	1.30
